# Enzyme systems of detoxication – overview of recent approaches

**DOI:** 10.2478/v10102-010-0022-2

**Published:** 2010-11

**Authors:** Pavel Anzenbacher, Eva Anzenbacherová

**Affiliations:** 1 Department of Pharmacology, Faculty of Medicine and Dentistry, Palacky University, CZ-775 15 Olomouc, Czech Republic; 2 Department of Medical Chemistry and Biochemistry, Faculty of Medicine and Dentistry, Palacky University, CZ-775 15 Olomouc, Czech Republic

The most important enzymes of detoxication are cytochromes P450 of Phase I and UDP-glucuronosyltransferases of Phase II of drug metabolism. The conventional division of drug, or xenobiotic, metabolism to two phases has survived almost fifty years and although not perfect, still is rather informative and practical. The recent addition of the next, third phase to the first two (Phase I as yielding a molecule with free functional groups ready for conjugation with another molecule or its part in Phase II) stressed the importance of drug transport across the membranes even if it is not a pure metabolic process (i.e. a process changing the polarity and structure of a compound).

The importance of testing the toxicity of newly invented drugs and drug candidates is also given by the simple fact that it is the toxicity and unwanted pharmacokinetics incl. interactions which is the reason for declination of one half of new or promising compounds. All other reasons as low efficacy, adverse effects, marketing and commercial reasons, and other reasons are responsible for the second half of the rejections (Van de Waterbeemd & Gifford, 2003). According to the recent view of the regulation authorities, the *in vitro* testing of drug metabolism of any new chemical entity should be realized together with an evaluation of its interaction with the system of drug transporters, mainly with the ABC membrane based protein pumps which often decide on the drug bioavailability.

The majority of known cases of drug toxicity are however ascribed to reactions in which the catalytic processes involving cytochromes P450 are involved. The death toll caused by malignant arrhythmias due to increased level of terfenadine or astemizole is well documented and is a part of each modern textbook of pharmacology and toxicology. Also, the recent case of mibefradil which has been withdrawn from the market by the company two years after successful introduction is also well known. In all cases, increased levels of the drug were caused by interactions with other drugs, competing for the same form of cytochrome P450. Cytochromes P450 are also known for their two-sided behavior often compared with Dr. Jekyll and Mr. Hyde of the R. L. Stevenson′s novel: The same form is responsible for detoxication processess as well as by activation of a carcinogen or a toxicant, in principle, by the same type of chemical reaction, i.e. by activation of molecular oxygen. It is of course not the fault of the cytochromes P450, but our own – as the enzyme is simply performing the same role independently on our wish.

The ways and approaches used for *in vitro* studies of processes in which cytochromes P450 take place can be divided according to the degree of organization of the system studied. From the most simple to the most complex, the first system should comprise only the molecules of the enzyme with the necessary components assuring the proper assembly and function.

## Reconstituted Systems, Bactosomes *et al*.

These so-called “reconstituted systems” with cytochromes P450 should at first include the studied form of cytochrome P450, the NADPH:cytochrome P450 oxidoreductase, NADPH as the source of electrons (or a system generating the NADPH as the NADP, isocitrate and isocitrate dehydrogenase or alternatively again the NADP, and glucose-6-phosphate together with glucose 6-phosphate dehydrogenase). Also, a phospholipid is necessary to form a moiety similar to the membrane of endoplasmic reticulum. Similarly, the reconstituted systems are prepared with specified forms of UDP-glucuronosyltransferase, or with the sulfotransferase or other enzymes to study the second phase of xenobiotic metabolism (Shimada & Yamazaki, 1998; Green & Tephly, 1998).

An alternative to these reconstituted systems is the use of membrane preparations of mostly bacterial origin expressing only a single form of cytochrome P450, but together with the NADPH:cytochrome P450 oxidoreductase supplying the necessary electrons. As with the reconstituted systems, a surplus of the NADPH or a NADPH generating system must be added. An advantage of these recombinant systems is certainly the fact, that the enzymes are embedded in the membrane and that no addition of a phospholipid as well as preparation of a vesicular system by ultrasound is needed (See e.g. web sites www.cypex.co.uk (bactosomes), www.bdbiosciences.com (supersomes), www.invitrogen.com (baculosomes)).

Studies with reconstituted systems and with membraneous systems containing single form of cytochrome P450 are complex as the composition and even the mode of preparation (e.g. sequence of components added) of the reaction mixture can significantly affect the efficacy of the system. On the other hand, these systems are the only way how to confirm that a particular, selected enzyme is involved in the biotransformation of the substance studied and that it forms the desired products (either more or less toxic or with unchanged toxicity).

## Microsomes

Microsomes, i.e. microsomal fraction of cell homogenate (most frequently liver microsomes formed from hepatocytes) are artificial systems formed during cell disruption and obtained by differential centrifugation (Schenkman & Jansson, 1998). The most important point is that the “microsomes” maintain the orientation of the endoplasmatic reticulum from which they are formed. Studies with microsomes need addition of NADPH or of the NADPH generating system as the amount of this coenzyme in the microsomal preparation is quickly depleted during the reaction. Liver microsomal enzymes are not only cytochromes P450, but also e.g. flavin monooxygenases, xanthin oxidase, quinone oxidoreductase, as well as several enzymes of the conjugation phase of xenobiotic metabolism as UDP glucuronosyltransferases (families UGT1A, UGT2B) and others. Hence, the reaction performed in this system yields products of several enzymes and the analysis is more complex. Hence, an inhibition of specific activities of selected enzymes should be used to find the enzyme system or a particular form of enzyme (e.g. of the cytochrome P450) which is involved in an interaction with the compound studied. This way, the “suspected” enzyme or enzyme form can be selected. Also, a reverse attitude can be used – when a metabolite is formed, the addition of a specific inhibitor of a particular enzyme can influence its formation (Jančová *et al*., 2007). The microsomes are by no means still the most widely used system in studies of xenobiotic metabolism and toxicity.

## Hepatocytes

Cellular systems, as liver hepatocytes, are more and more frequently used. The commercial availability of hepatocytes from defined sources makes their use rather easy. In this respect, freshly thawn cells according to the supplier's recommendation are the best solution for laboratories which do not have access to cell culture laboratory. Alternatively, a freshly prepared suspension of hepatocytes can be used for limited time (not exceeding two hours) after disruption of the organ. Hepatocytes itself represent even more complex system than microsomes which however mimic well the situation on the organism (Ferrini *et al*., 1998).

The most of prime selection and pilot toxicological studies usually stop at this degree of complexity. For human use, a toxicity study on model experimental animal is still necessary. The realization of such an experiment and the selection of an appropriate model are however beyond the scope of this introduction.

## References

[CIT0001] Van de Waterbeemd H, Gifford E (2003). ADMET in silico modelling: towards prediction paradise?. Nature Revs Drug Discovery.

[CIT0002] Shimada T, Yamazaki H (1998). Cytochrome P450 reconstitution systems. Meth Mol Biol.

[CIT0003] Green MD, Tephly TR (1998). Glucuronidation of amine substrates by purified and expressed UDP-glucuronosyltransferase proteins. Drug Metab Dispos.

[CIT0004] Schenkman JB, Jansson I (1998). Isolation and purification of constitutive forms of microsomal cytochrome P450. Meth Mol Biol.

[CIT0005] Jančová P, Anzenbacherová E, Papoušková B, Lemr K, Lužná P, Veinlichová A, Anzenbacher P, Šimánek V (2007). Silybin is metabolized by cytochrome P450 2C8 *in vitro*. Drug Metab Dispos.

[CIT0006] Ferrini JB, Ourlin JC, Pichard L, Fabre G, Maurel P (1998). Human hepatocyte culture. Meth Mol Biol.

